# Monitoring for Asbestos: U.S. EPA Methods

**DOI:** 10.1289/ehp.112-a728b

**Published:** 2004-09

**Authors:** Kathleen C. Callahan

**Affiliations:** U.S. Environmental Protection Agency, Region 2, New York, New York, E-mail: callahan.kathy@epa.gov

I would like to correct a misimpression about the methods used by the U.S. Environmental Protection Agency (EPA) in monitoring for asbestos in the air following the collapse of the World Trade Center in “Health and Environmental Consequences of the World Trade Center Disaster” (Landrigan et al. 2004). The authors state that

More than 10,000 ambient air samples from lower Manhattan were tested for asbestos by the U.S. EPA using phase-contrast light microscopy (PCM) to identify fibers > 5 mm in length; more than 8,000 of these samples were also examined by transmission electronic microscopy (TEM) to identify fibers of ≥0.5 mm in length.

This suggests that the U.S. EPA placed more emphasis on the analysis of asbestos in air samples using phase-contrast light microscopy (PCM) than those examined by transmission electron microscopy (TEM). This is not the case.

Recognizing the potential asbestos hazard, the U.S. EPA initiated its asbestos environmental sampling on the afternoon of September 11, employing TEM analysis as the primary method of recording the presence of asbestos fibers. The agency relied more heavily on the TEM data because PCM analysis cannot distinguish asbestos from other mineral fibers and would therefore not provide as accurate a measure of airborne asbestos concentrations as TEM.

As directed in the procedures outlined in the Asbestos Hazard Emergency Response Act (AHERA) (U.S. EPA 1987), TEM counts were recorded for both short (0.5–5 mm) and long (> 5 mm) asbestos fibers. The U.S. EPA’s World Trade Center website (U.S. EPA 2004) summarizes the results of 9,604 asbestos samples from 22 monitoring stations in lower Manhattan that were analyzed by TEM, not the 8,000 samples cited in the article (Landrigan et al. 2004).

Most of the asbestos samples were also analyzed by PCM. The PCM analysis was performed to provide ancillary information about total fiber counts and data for the Occupational Safety and Health Administration.

Because there has been much public confusion about the use of the two analytic methods in the World Trade Center response, I felt it was especially important to correct and clarify that the U.S. EPA used the most accepted and appropriate method to protect the health of residents and response workers in the aftermath of the disaster.
